# Supplementary material for: Exploring spatial variations and the individual and contextual factors of uptake of measles-containing second dose vaccine among children aged 24 to 35 months in Ethiopia
Source: PLoS One. 2023 Jan 4;18(1):e0280083. doi: 10.1371/journal.pone.0280083 (PMC9812309; doi:10.1371/journal.pone.0280083)
Supplement: Attachment — Submitted filename: Spatial distribution and factors associated with uptake of measles-containing second dose vaccine among children aged 24 to 35 months in Ethiopia.docx [file pone.0280083.s001.docx]

Review for PLOS ONE 28.01.2022

Paper tile: **Spatial distribution and factors associated with uptake of measles-containing second dose vaccine among children aged 24 to 35 months in Ethiopia**

**FEEDBACK**

**General feedback**

This manuscript has the potential to further contribute to understanding MCV2 vaccination inequalities in Ethiopia and ts findings could be used by policy makers to inform interventions aiming to improve MCV2 vaccination coverage in Ethiopia. The subject (MCV2 coverage) is of interest to many and can enrich the available literature on understanding MCV2 coverage determinants when published. The methods used are appropriate for the study. However, the manuscript requires a major revision including language editing before it will be suitable for publication. The data source for the manuscript seems inappropriate because data collection was started just one month after the introduction of MCV2 thereby likely biasing the true MCV2 coverage downwards.

**Specific feedback**

I think, at this stage, grouping my feedback into different themes would be more efficient than giving it line by line.

***Language***

This manuscript requires language editing to be suitable for publication.

***Background***

1. Mention the agenda referred in line 102.
2. I suggest reframing the objective (lines 103 – 104) to something like “…. to explore individual and contextual factors associated with the uptake of the second dose of a measles-containing vaccine among children aged 24 – 35 months in Ethiopia”.

***Methods***

1. Lines 109 – 110: Considering that routine MCV2 was introduced in Ethiopia in February 2019 and the data collection for the EMDHS (data source for this study) was conducted arch to June of the same year, it is possible that not all the children in the study sample had access to the vaccine before the data collection period. I suspect that this data source is inappropriate for objectives of this study as the time between the introduction of the vaccine and data collection are very close. Please indicate if the MCV2 dose was introduced through a campaign approach or otherwise. If it was not introduced trough a campaign approach, this limitation of the data source should be stated in the discussion/methodological considerations. If the vaccine was introduced in different regions in different dates, this should lo be indicated as it can also influence the coverage.
2. Line 129: I think the sample size (unweighted = 800 and weighted = 809) is referring to children 24 – 35 months not their mothers since it is the children’s dataset which is used.
3. Lines 136 – 142 (independent variables): I recommend mentioning how each variable was operationalized/categorized instead of the three indicated.
4. Lines 144 – 149 – Operational definition for region giving here is not the same as the one used in the spatial analysis. Going by the re-categorization explained here, one would expect a similar merging on the maps presented.
5. Lines 151 -155: Usually, the definition for full vaccination for basic vaccines is 1 dose of BCG, three doses of DPTCV/Penta, 3 doses of OPV, and 1 dose of MCV. I suggest using this definition since it is the same definition used in the EMDHS as can read from the report using the link below <https://dhsprogram.com/pubs/pdf/FR363/FR363.pdf>.
6. Line 161 and elsewhere: I suggest replacing “proportion of uptake of MCV2” with something like “MCV2 uptake” or “MCV2 coverage” or “proportion of children vaccinated with MCV2”, etc.
7. Lines 170 -171: I think this approach to variable selection is perhaps more useful for building predictive models. Though it is widely used, I think is better to include variables based on a priori and their availability in the dataset in this case because the significance level or effect of an independent variable in bivariable analysis may not be exactly the same when introduced in a multivariable regression model.
8. Lines 203 – 204: Perhaps it will be better to just mentioned that you used a secondary dataset and whether the EMDHS was ethically cleared.

***Results***

Line 221: Since vaccination card retention was reported to be low among the respondents for this study (26% - <https://dhsprogram.com/pubs/pdf/FR363/FR363.pdf>), I recommend doing a sensitivity analysis to assess the potential recall bias and report the results here and in the discussion.

***Discussion***

1. I suggest restructuring the discussion in the following order:
   a. Summarize the key findings of the study in relation to the study objective.
   b. Interpret (explain) the results taking into account the study objective, how the results compare with similar studies, etc.
   c. Highlight the policy and research implications of the study.
   d. Discuss the strengths and weaknesses of the study.
2. You may wish to consider including the following methodological limitations in your discussion:
   a. The short time span between MCV2 introduction and the beginning of data collection for the EMDHS.
   b. The potential of recall bias (depending on the results of the sensitivity analysis) considering the low card retention rate in the age cohort used in this study.
3. The explanation given in lines 289 – 292 for older mothers seems to be in conflict with the one given in lines 293 – 295 birth order number assuming that older mothers will generally have more children than younger mothers.

***References***

You may wish to consider the following points (they can be easily fixed):

1. Some of the references seem to be inappropriate where they are cited. For example,
   a. References 8 & 9 would be better placed where reference 6 & 7 are because they are studies about inequalities in coverage but not deaths.
   b. Reference 10 is about the prevalence of anemia but it is cited for how measles is spread.
2. Some references seem a bit too old to use. For example:
   a. The refences (25 & 26) for MCV2 coverage in Africa are outdated in the sense that coverage has improved a lot now compared to the years referred (2014). That was at a time that many African countries had not introduced the second dose of the MCV2 dose.
